# Reduction of Real-Time Imaging of M1 Macrophage Chemotaxis toward Damaged Muscle Cells is PI3K-Dependent

**DOI:** 10.3390/antiox7100138

**Published:** 2018-10-08

**Authors:** Hiromi Yano, Masataka Uchida, Tatsuya Saito, Takafumi Aoki, Michael J. Kremenik, Eri Oyanagi

**Affiliations:** 1 Department of Health & Sports Science, Kawasaki University of Medical Welfare, 288 Matsushima Kurashiki, Okayama 701-0193, Japan; t.saito@mw.kawasaki-m.ac.jp (T.S.); kremelin@mw.kawasaki-m.ac.jp (M.J.K.); eri-oyanagi@mw.kawasaki-m.ac.jp (E.O.); 2 Graduate School of Health Science and Technology, Kawasaki University of Medical Welfare, Okayama 701-0193, Japan; aoki.takafumi.54331@mw.kawasaki-m.ac.jp; 3 Faculty of Sport and Health Science, Ritsumeikan University, Shiga 525-8577, Japan; m-uchida@fc.ritsumei.ac.jp

**Keywords:** J774, C2C12, TAXIScan, lipopolysaccharide, Ly294002

## Abstract

Macrophages migrate and invade into damaged muscle rapidly and are important for muscle repair and subsequent regeneration. The exact cellular and biological events that cause macrophage migration toward injured muscle are not completely understood. In this study, the effect of macrophage differentiation on the chemotactic capability to invade local damaged muscle was investigated using an in vitro model of muscle injury. We used C2C12 cell myoblasts and J774 cell macrophages, and the “killed-C2C12” cells were combined with live C2C12 cells as a partially damaged muscle model. The cultured J774 cells, with or without lipopolysaccharide (LPS), were treated with Ly294002 (Ly), which is an inhibitor of phosphoinositide 3-kinase (PI3K). In order to evaluate the polarization effect of LPS stimulation on J774 cells, expression of cell surface Toll-like receptor 4 (TLR4), CD11c and CCR2, and expression of F-actin intensity, were analyzed by flow cytometry. The real-time horizontal chemotaxis assay of J774 cells was tested using the TAXIScan device. The expressions of TLR4, CD11c, and F-actin intensity in LPS-treated cells were significantly higher than those in Ctrl cells. In LPS-treated cells, the chemotactic activity toward damaged muscle cells completely disappeared. Moreover, the reduced chemotaxis depended far more on directionality than velocity. However, Ly treatment reversed the reduced chemotactic activity of the LPS-treated cells. In addition, cell-adhesion and F-actin intensity, but not CCR2 expression, in LPS-treated cells, was significantly reduced by Ly treatment. Taken together, our results suggest that the PI3K/Akt activation state drives migration behavior towards damaged muscle cells.

## 1. Introduction

It is known that macrophages as well as neutrophils migrate and infiltrate into the location of damaged muscle [1,2,3]. This immunological response is considered to be important for muscle repair and subsequent regeneration [4,5,6,7,8,9].

The phenotype of macrophages, which display different functional features, influences chemotaxis [10]. Macrophage phenotypes were classified into two groups: Classically activated macrophages (M1) and alternatively activated macrophages (M2), which are further divided into groups M2a, M2b and M2c [11]. Although lipopolysaccharide (LPS), which strongly induces differentiation into inflammatory macrophages such as M1, also affects macrophage motility, the effects of macrophage differentiation on chemotaxis toward damaged muscle has been poorly understood.

Kleveta et al. (2012) [12] showed that F-actin expression was upregulated by LPS treatment, and moreover, we reported that LPS-treated macrophages were accompanied by both a high F-actin expression with a low expression of CCR2 (a receptor for monocyte chemoattractant protein-1) and a decrease in chemotactic activity [13]. Therefore, macrophage differentiation strongly influenced the chemotactic activity toward damaged myoblast cells through the expression of F-actin [13] and CCR2 [13,14].

Inflammation, such as that induced by LPS stimulation, upregulates the phosphoinositide 3-kinase (PI3K)/Akt signaling pathway [15,16]. In addition, the possible relevance of mTOR/S6K, which is present downstream of the PI3K/Akt signaling pathway, in neutrophil migration, has been shown [17]. The important role of the PI3K/Akt signaling pathway in the regulation of stress fiber formation and cell migration is already known [18,19,20]. Moreover, activation of the PI3K/Akt signaling pathway is essential for macrophage migration [21], as well as other migratory cells. Hence, when cells need to negatively regulate migration, F-actin remodeling up-regulates and cell adhesion increases. A recent report showed that, with LPS stimulation, one of the Rab family of small GTPases recruits PI3K, which regulates Akt signaling generated by surface Toll-like receptor 4 (TLR4) on macrophages, and then regulates mTOR signaling in macrophages [22]. It seems that this signal pathway regulates multiple steps of membrane trafficking and phagocytosis of pathogens [22]. However, it remains unclear whether LPS-induced PI3K activation is associated with inflammatory macrophage chemotaxis toward damaged muscle cells.

In this study, we investigated the effect of macrophage differentiation on the real-time imaging of inflammatory macrophage chemotaxis toward damaged muscle cells. Our hypothesis was that macrophage chemotaxis toward damaged muscle cells influences their differentiation through the activation of PI3K.

## 2. Materials and Methods

### 2.1. Reagents

Lipopolysaccharide (*Escherichia coli* 055:B5) and Ly294002 (Ly) were purchased from Sigma (St Louis, MO, USA). 

### 2.2. Cell Culture

Mouse myoblasts cell line C2C12 cells and mouse macrophage cell line J774 cells were purchased from the Cell Bank Riken Bioresource Center (Ibaraki, Japan). These cells were grown in a 10-cm dish with Dulbecco’s Modified Eagle’s medium containing 10% heat-inactivated FCS, added with 200 U/mL penicillin and 100 µg/mL streptomycin, at 37 °C in 5% CO_2_.

In all experiments, the C2C12 cells were switched to a 6- or a 96-well flat-bottomed cell culture plate after they reached the myoblast stage. The medium was changed the day after seeding and each second day thereafter. The C2C12 cells were used from passage 2 to 10. The killed-C2C12 cells were induced by repeat freezing (three times) by liquid nitrogen for 60 sec and incubation at 37 °C for 3 min, and then they were combined with live C2C12 at 1:0.5 (live:killed C2C12) as a partially damaged muscle model [13].

J774 cells were stimulated by 100 ng/mL LPS with or without 20 µM Ly for 24 h. The cells’ polarization was evaluated by the expression of surface TLR4 and CD11c using flow cytometry. Moreover, to evaluate M1 activation status, the concentration of tumor-necrosis factor (TNF)-α in the cells-conditioned medium sample was measured 24 h after the stimulation.

### 2.3. Real Time Horizontal Chemotaxis Assay Using TAXIScan

Chemotaxis assays of J774 cells were carried out using the TAXIScan device (ECI, Tokyo, Japan) [13,23,24]. Migration of J774 cells at 37 °C was recorded every 3 min. The migrating cells were traced by clicking each cell on the display and the average values of parameters were calculated. The data were from three independent observers. The velocity of cell migration is expressed µm/s. The directionality of migration is expressed as the angle (radian) toward the opposite compartment (i.e., π/2 indicates that the J774 cell is migrating toward the C2C12 cells).

#### 2.3.1. Flow Cytometric Analysis

The expression of surface TLR4, CD11c and CCR2 on each macrophage was measured by flow cytometric analysis. Briefly, the cells (2 × 10^5^ cells) were treated with FcR blocker (mouse normal serum, Biomeda, Foster City, CA, USA) for 30 min on ice, and then stained in ice-cold PBS containing 0.3% BSA and 0.05% NaN_3_ with the following antibodies: Anti-TLR4-PE, anti-CD11c-FITC and isotype controls (BD Bioscience, San Diego, CA, USA), and CCR2 (anti-mouse CCR2 rabbit polyclonal antibody, Abcam, Cambridge, UK). F-actin was stained by phalloidin (Acti-stain™ 555 phalloidin, Cytoskeleton, Denver, CO, USA). The flow cytometric analysis was performed using the FACSCalliber™ Flow Cytometry System with the CellQuest software package (BD, Franklin Lakes, NJ, USA). The change in the mean fluorescence intensity (MFI) between anti-TLR4 and CD11c antibodies and each isotype control were obtained to quantify the expression of each marker.

#### 2.3.2. Adherence to Plastic

J774 cells (2 × 10^5^) were seeded into untreated plastic flat-bottomed (96-well) plate and incubated at 37 °C for 60 min. Then, we removed the non-adherent cells and then fixed the remaining cells with 2% glutaraldehyde for 10 min. The cells were stained with 0.5% crystal violet in 200 mM boric acid for 15 min after wells were washed twice with H_2_O. After they were washed three times, the cells were lysed by 10% acetic acid and measured OD by 560 nm.

#### 2.3.3. Western Blot Analysis

Cells, which were washed in PBS, were lysed by RIPA buffer with a protease inhibitor cocktail (25955-11, Nacalai Tesque, Tokyo, Japan), and then their protein concentrations were measured by Bradford assay (Bio-Rad, Hercules, CA, USA). Equal amounts of extracts were separated by 10% SDS-PAGE, and then were transferred onto a nitrocellulose membrane, which was probed with primary antibodies overnight at 4 °C. After incubation with HRP-conjugated secondary antibodies at room temperature for 60 min, the target proteins were detected with a chemiluminescence kit (Chemi-Lumi One Super, 02230, Nacalai Tesque), and quantified with Amersham Imager 600 (GE Health Care, Chalfont St. Giles, UK). Primary antibodies used include those against Akt (#9272, 1:1000), phospho-Akt (#9275, 1:1000) and GAPDH (#2118, 1:10,000) from Cell Signaling Technology (Danvers, MA, USA), which was used as an internal control.

#### 2.3.4. Enzyme-Linked Immunosorbent Assay (ELISA)

The concentration of TNF-α was measured by ELISA (a murine and human kit, R&D Systems, Minneapolis, MN, USA). The absorbance was measured at 450 nm and was proportional to the concentration of TNF-α in the sample.

### 2.4. Statistical Analysis

Data were expressed as mean ± SEM. Statistical calculations were carried out with the SPSS 15.0 for Windows software program. The data were analyzed using the one-way analysis of variance (ANOVA). Bonferroni’s tests were performed as post-hoc. A p-value less than 5% was considered statistically significant.

## 3. Results

### 3.1. Effect of Ly Treatment on LPS-Induced Differentiation on the Macrophages

**Figure 1:**
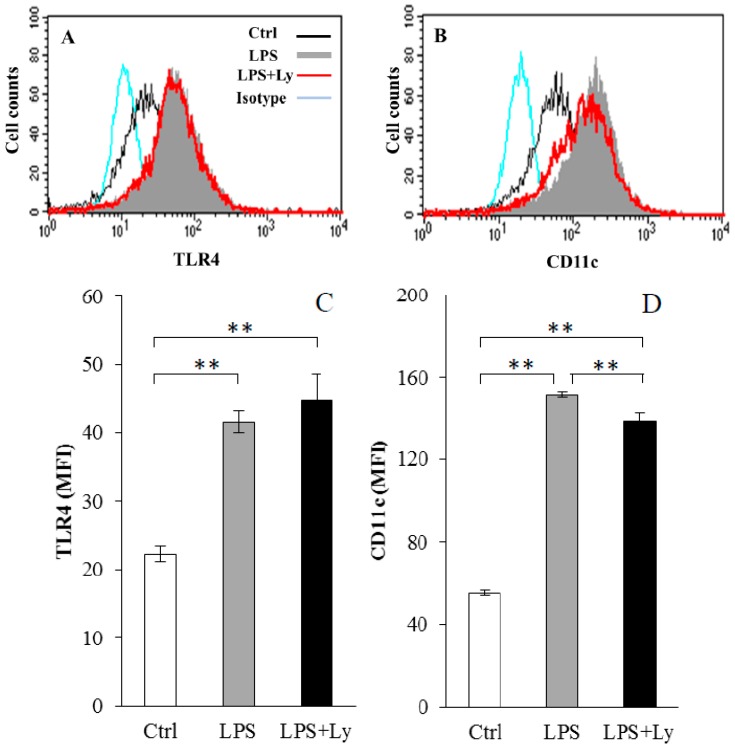
The cell-surface expression of Toll-like receptor 4 (TLR4) (**A**,**C**) and CD11c (**B**,**D**) on J774 cells after lipopolysaccharide (LPS) and/or Ly treatments. Typical data (**A**,**B**) and MFI (the mean ± SEM (**C**,**D**)) of LPS and LPS+Ly treated J774 cells are shown (*n* = 6). ** *p* < 0.01.

The cell-surface expression of TLR4 and CD11c on J774 cells after LPS and/or Ly treatments are shown in Figure 1. Expressions of TLR4 and CD11c on cell surface in both LPS- and LPS+Ly-treated cells were significantly higher than that in Ctrl cells (*p* < 0.01, Figure 1C and D). Although the expression level of TLR4 between LPS and LPS+Ly cells showed no significant difference (Figure 1C), CD11c expression on LPS+Ly cells was significantly lower than that on the LPS cells (*p* < 0.01, Figure 1D). 

### 3.2. Effect of Ly Treatment on LPS-Induced Differentiation in the Macrophages

**Figure 2:**
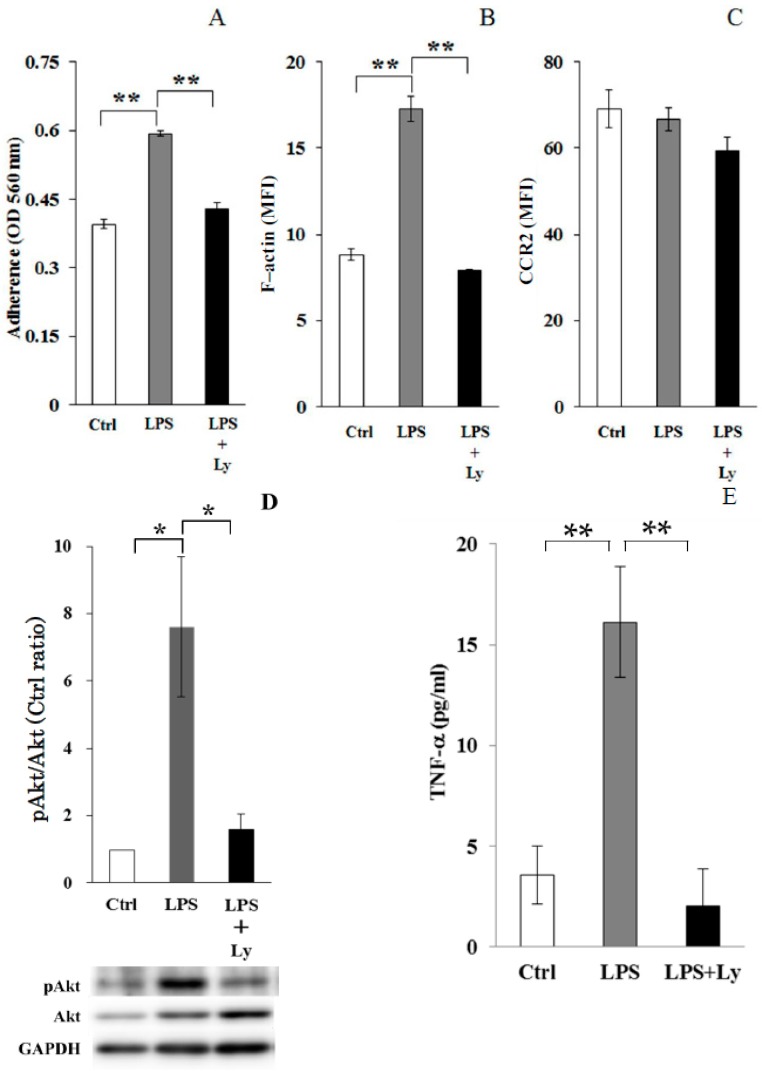
The effect of phosphoinositide 3-kinase (PI3K) inhibitor Ly treatments on cell adherence (**A**), F-actin (**B**), CCR2 (**C**), phosphorylation of Akt proteins (**D**) and TNF-α production (**E**) of J774 cells (*n* = 8). * *p* < 0.05 and ** *p* < 0.01. Western blot results of phospho-Akt (pAkt)/total Akt. Results were quantitated by densitometry and ratios of pAkt/total Akt were plotted. Y axis is in arbitrary unit (**D**).

Cell adherence, which was significantly increased by LPS stimulation (*p* < 0.01, vs. Ctrl cells), was strongly inhibited by Ly treatment (*p* < 0.01, vs. LPS cells, Figure 2A). F-actin intensity also had the same result as cell adherence (Figure 2B). However, the influence of LPS and Ly stimulation on CCR2 expression was not observed (Figure 2C).

The high level of phosphorylation of Akt (pAkt)/total Akt seen in LPS treated cells (*p* < 0.05, vs. Ctrl cells) was significantly inhibited by Ly treatment (*p* < 0.05, Figure 2D). Additionally, increasing TNF-α production in LPS-treated cells was also inhibited by Ly treatment (*p* < 0.01, Figure 2E).

### 3.3. Effect of Ly Treatment on LPS-Induced Differentiation on the Cells Chemotactic Activity

**Figure 3:**
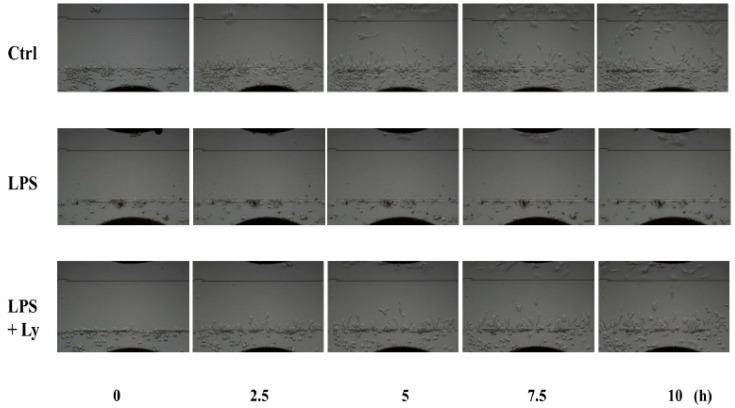
Direct comparison of 5 frames from the video (selected frames in order from 0 h to 10 h) with equivalent frames from the chemotaxis of macrophages toward damaged C2C12 cells.

**Figure 4:**
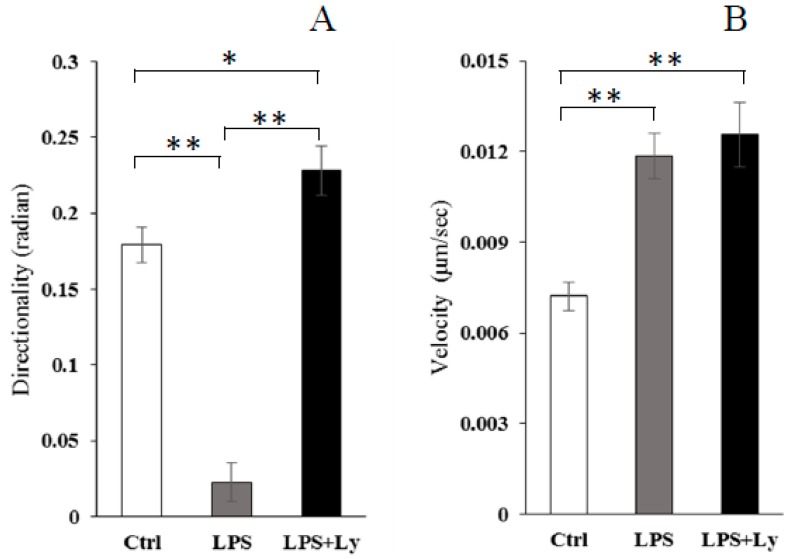
Chemotaxis, shown as directionality (**A**) and velocity (**B**), of J774 cells toward damaged C2C12 cells (*n* = 100 for each condition). * *p* < 0.05 and ** *p* < 0.01.

Direct comparison of 5 frames from the video (Video S1) with equivalent frames from the chemotaxis of macrophages toward damaged C2C12 cells is shown in Figure 3. Although chemotaxis of Ctrl cells toward damaged C2C12 cells was observed, that of LPS-treated cells disappeared completely. However, this disappearance of chemotaxis was not observed in the case of LPS+Ly-treated cells. Moreover, in this experiment, chemotaxis of macrophages was evaluated in each directionality and velocity (Figure 4). The directionality of LPS-treated cells toward C2C12 cells was significantly lower than that of both Ctrl and LPS+Ly cells (*p* < 0.01, respectively), and then the directionality of LPS+Ly cells is slightly, but significantly, higher than that of Ctrl cells (*p* < 0.05, Figure 4A). On the other hand, the velocity was not different between LPS+Ly and LPS cells, although the velocity of both LPS+Ly and LPS cells was significantly higher than that of Ctrl cells (*p* < 0.01, respectively, Figure 4B).

## 4. Discussion

This study demonstrates that LPS-inhibited macrophage chemotaxis to invade local damaged muscle is associated with F-actin expression, and the inhibition of macrophage chemotaxis was completely reversed by the PI3K inhibitor. These findings suggest that macrophage chemotaxis toward damaged muscle depends on their differentiation through the activation of PI3K.

In this study, a directed migration experiment was performed using a TAXIScan device assay. TAXIScan is an assay device for studying cell dynamics in vitro, which functions by providing two-dimensional images of cell migration. Accordingly, this device can provide markedly more information, including morphology as well as quantitative analysis, compared to existing methods such as the Boyden chamber method [13,24,25,26]. Interestingly, the directionality and velocity of chemotactic cells can be provided by analyzing the cell images. In fact, we observed that the directionality of J774 cells toward damaged muscle cells was inhibited by LPS treatment, and that it was completely rescued by Ly treatment. In contrast, the velocity of J774 cells was not different between LPS and LPS+Ly cells. A previous study reported that the chemotactic activity of M1 macrophages to damaged skeletal muscle cells is lower than that of M2 macrophages, which is more directionally dependent than velocity [13]. Expressions of *Cd11c* and *Tlr4* mRNA as markers of pro-inflammatory M1 macrophages have been demonstrated [24,27]. Thus, our results are strongly supported by this previous study. In addition, Uchida et al. (2013) [13] suggested that the low chemotactic activity of M1 macrophages toward damaged muscle cells is associated with a high expression of F-actin and a low expression of CCR2. F-actin expression is upregulated by LPS treatment [4]. Moreover, it was shown that the distribution of F-actin in LPS-treated cells was observed strongly around the cell membrane, when the expression of F-actin in macrophages was increased by LPS stimulation [13]. Since F-actin remodeling has an essential function in cell movement and cell adhesion [28], we tried to examine the influence of F-actin expression in LPS-treated macrophage chemotaxis. As for the results, Ly treatment inhibited LPS-induced F-actin expression when the reduced chemotaxis of macrophages was rescued by the Ly treatment. LPS stimulates the PI3K/Akt signal, up-regulates F-actin remodeling and also increases cell adhesion [11]. Moreover, it has been suggested that TLR4 signaling involves PI3K activation [11]. Therefore, movement in one direction, such as the direction toward damaged myoblast cells, might be limited by F-actin remodeling, which is induced by the LPS/TLR4-PI3K/Akt pathway.

On the other hand, chemokines are the major mediators of macrophage chemotaxis via chemokine receptors. The low chemotactic activity of M1 macrophages toward damaged muscle cells might be associated with expression of CCR2. However, in the present study, the influence of LPS and Ly stimulation on CCR2 expression on macrophages was not observed, although chemotaxis was greatly different between LPS and LPS+Ly stimulations. Also, in previous studies, LPS induced a reduction [29,30], no effect [25] or an increase [26] in CCR2. This discrepancy might be attributable to different assay conditions or cells used. Thus, the matching results are not shown. At least, our results strongly suggest that macrophage chemotaxis toward damaged muscle depends on their degree of F-actin remodeling through the activation of PI3K. It seems that M1 macrophages are thought to migrate over shorter distances compared with M2 macrophages. Our previous study, and other studies, showed that M2 macrophages, compared to M1 macrophages, displayed enhanced motility towards damaged muscle cells and chemokines (CCL2, CXCL12 and C1q), respectively [13,25].

Our study has limitations. Firstly, LPS+Ly macrophages showed constant expression of TLR4 and CD11c, which are M1 macrophage markers, on the cell surface. However, PI3K inhibition by Ly treatment results in a robust reduction of TNF-alpha release. Therefore, it is possible that LPS+Ly macrophages are no longer of the M1 phenotype. In fact, it was already reported that Ly treatment induces suppression of TNF-α secretion [15,16]. Although a high expression of CCR2 on the cell surface of M2 cells is known [13], the PI3K inhibitor did not affect the CCR2 expression on the LPS-treated cells. Furthermore, a recent study suggested that CCR2 on macrophages was associated with M1-phenotype switching in addition to cell migration [31]. In our study, the CCR2 expression showed no significant difference between LPS and LPS+Ly macrophages. Because PI3K exists as an upstream event of the NF-κB pathway, which is the important downstream pathway mediated by TLR4 [15,16], PI3K inhibitor Ly treatment induced this paradoxical phenomena, which means that it inhibited TNF-α secretion despite constant expression of TLR4 and CD11c on the cell surface.

Secondly, this study cannot answer the point, which Perdiguero et al. (2011) [32] made, that the PI3K/Akt inhibitor disturbs normal macrophage cell behavior in injured muscle over time and could also delay cytokine silencing during the resolution of inflammation. Therefore, further study is needed as to whether regulation of inflammation-mediated tissue repair is possible by continuously controlling PI3K/Akt activation. 

In conclusion, we investigated the effect of macrophage differentiation on the real-time imaging of inflammatory macrophage chemotaxis toward damaged muscle cells. Our results suggest that the PI3K/Akt activation state drives migration behavior towards damaged muscle cells.

## Supplementary Materials

The following are available online at http://www.mdpi.com/2076-3921/7/10/138/s1, Video S1: A video showing the macrophages chemotaxis toward C2C12 cells (live:killed cells = 1:0.5). Ctrl: unstimulated-J774 cells, LPS: LPS-treated J774 cells and LPS+Ly: Ly treated LPS-J774 cells. In each picture, C2C12 and J774 cells seeded on the upper and the lower compartments, respectively.
